# Upper limb motor recovery in chronic stroke—longitudinal aggregate analysis from control group outcomes

**DOI:** 10.3389/fresc.2025.1448174

**Published:** 2025-08-22

**Authors:** Fabien Scalzo, Robert A. Coker, Lauren Souders, Leo Petrossian, Kern Bhugra, Lauren Sheehan, Eric C. Leuthardt, Alexander R. Carter

**Affiliations:** ^1^ Keck Data Science Institute, Pepperdine University, Malibu, CA, United States; ^2^ Kandu, Inc., Los Angeles, CA, United States; ^3^ Department of Neurological Surgery, Washington University School of Medicine, St. Louis, MO, United States; ^4^ Department of Neurology, Washington University School of Medicine, St. Louis, MO, United States

**Keywords:** stroke, rehabilitation, Fugl-Meyer assessment, motor recovery, upper extremity

## Abstract

**Introduction:**

This study examines the effects of regular physical activity on upper extremity motor recovery during the late subacute and chronic phases of stroke.

**Methods:**

Data were aggregated from 20 studies comprising 368 participants in control groups receiving usual care or general rehabilitation without specialized interventions. To isolate the impact of non-specific physical activity, studies involving robotics or task-specific therapies were excluded.

**Results:**

The primary outcome was the change in Fugl-Meyer Assessment of Upper Extremity (FMA-UE) motor scale. The pooled effect size for FMA-UE change was small and non-significant (Cohen's *d* = 0.11, 95% CI: −0.05 to 0.26, *p* > 0.05), indicating that general physical activity alone may result in limited improvements in upper extremity function in chronic stroke. Heterogeneity across studies was low, and no evidence of publication bias was found.

**Discussion:**

These findings provide a quantitative benchmark for expected gains from general activity and offer a reference for interpreting outcomes in future stroke rehabilitation trials lacking control groups.

## 1 Introduction

Regular physical activity and exercise have the potential to play a positive role in the recovery of persons with chronic impairments after stroke. Several studies have provided evidence that exercise in the acute and subacute recovery phases after stroke can improve cardiovascular fitness (1), walking ability (2), and upper-extremity muscle strength (3). Similarly, the improvements attributed to exercise extend beyond physical function and may improve depression (4), executive functioning and memory (5), and quality of life (6). Studies have also reported similar benefits in the chronic phase of recovery, with more variability about their magnitude. As such, general physical activity, which must be distinguished from targeted, mechanism-specific activity-based therapies such as constraint-induced movement therapy (CIMT) or task-specific training, may contribute to modest improvements even without targeted therapy. Therefore, some studies investigating the efficacy of a new specific activity-based intervention for chronic stroke may compare the treatment group to a control group receiving various other forms of physical activity to account for the non-specific effects of general physical activity itself. However, many pilot rehabilitation studies are limited to a single participant group and use a pre-post design due to logistical, financial, and time pressures that preclude the recruitment of a contemporaneous control group. Also, potential participants are less likely to enroll in a randomized controlled trial where they might be assigned to the control group, especially if the control treatment is not expected to produce benefit (absence of clinical equipoise). These challenges can limit the interpretability of positive results, which cannot be conclusively attributed solely to the specific intervention without a non-intervention group that also controls for the general effects of physical activity.

This study aggregates the motor assessment data reported in the literature from the control arm of studies investigating specific interventions in the chronic stroke population. The current analysis of the control group literature provides insight into the longitudinal changes of upper extremity motor status expected among persons with chronic impairments after stroke who engaged in moderate amounts of physical activity. In this study, we considered regular exercise and physical activity as those exercises aimed at increasing strength, endurance, balance, and coordination. We also incorporated physical activity related to incidental daily movements in our analysis. These activities are inherently more variable and lack a standardized structure. To ensure inclusivity, we did not impose any minimum thresholds for duration or intensity on these physical activities. However, we excluded any reported data that included technology assistance such as robotics devices or other formalized interventions such as task-specific training, massed practice, or constraint-induced movement therapy to reduce additional confounding factors and isolate the effect of general physical activity.

Our study is motivated by the desire to quantify the effect size of benefits derived from regular physical activity and exercise during the chronic phase of stroke recovery. This analysis can serve as preliminary evidence to support the need for a prospective meta-analysis. If confirmed, these results could be used as a reference for intervention studies lacking a control group to help assess if effect sizes significantly differ between intervention and general physical activity.

Much uncertainty exists regarding the role of general physical activity in motor recovery. We focus here on the benefits of upper extremity motor skills. For example, while aerobic exercise (7) in chronic stroke has demonstrated improvements in blood pressure, energy expenditure, and Vo2 max, whether these extend to upper extremity motor function remains unclear.

When considering the control group in the chronic phase of stroke recovery, the margin of improvement is highly variable; results reported by individual studies range from no benefits to significant improvement. Therefore, our study addresses the problem of quantifying the reported benefits into an aggregate that could be used as a more representative reference for new interventional studies.

In clinical studies of stroke recovery, the assessment of motor functions in patients is performed using standardized outcome measures. The most frequently used measures for assessing upper extremity impairment and activity capacity in stroke include the Fugl-Meyer Assessment of Upper Extremity (FMA-UE or FM), Action Research Arm Test (ARAT), Wolf Motor Function Test (WMFT), Box and Block test, Chedoke Arm and Hand Inventory, Nine Hole Peg Test, Modified Ashworth Scale (MAS), and Motor Status Scale (MSS). Although none of these metrics is perfect, these scales are generally considered reliable and responsive to motor function changes. They are also recommended as endpoints in stroke recovery trials. Recent studies have compared assessment tools, including the FMA-UE scale, to determine the optimal method to evaluate post-stroke impairment and demonstrated its reliability, validity, and sensitivity to treatment-related change (8–12). In addition, FMA-UE was reported as the most frequently used scale in a recent systematic review (13). These results support our choice of the FMA-UE scale as the primary outcome of the present study.

In this paper, we conduct an aggregate analysis to evaluate whether available evidence demonstrates changes in upper extremity motor status when a program of general physical activity is initiated at least 3 months after stroke.

Typically, the chronic phase of stroke is defined as beginning 6 months post-onset. According to the proportional recovery model (14, 15), up to 78% of the recovery in upper extremity function occurs within this 6-month period. Extending this model, recent evidence presented by Grefkes et al. (16) suggests that patients with mild initial deficits may reach a plateau in motor recovery earlier than 6 months. To maximize the number of studies eligible for inclusion in our analysis, we extended the inclusion threshold to 3 months post-stroke. Consequently, the patient populations analyzed may fall within what is typically defined as the late subacute phase, during which spontaneous recovery may still occur and may not be entirely attributable to physical activity.

Relevant studies published between 2000 and 2020 were identified via a search on PubMed. Inclusion criteria for this data analysis included patients in a control arm of a stroke trial who were treated with study-provided general physical activity initiated >3 months post-stroke; the study also was required to have collected scores on FMA-UE serially, i.e., before and after the provided physical activity program. Effect sizes for each study's control arm serial difference in FMA-UE (ΔFM) are aggregated in the data analysis. Next, a pooled effect size is computed using the standardized inverse variance weighting scheme. Here, the effect size quantifies the benefits associated with training persons with chronic impairments after stroke who are part of the control arm of the identified trials.

To provide some context, the minimal clinically important difference (MCID) in the subacute and chronic phases of stroke recovery are typically estimated around ΔFM = 9–10 points (17), and ΔFM = 4.25–7.25 points (18), respectively. These values are useful benchmarks when interpreting the efficacy of rehabilitation treatments.

The presence of publication bias and heterogeneity is also evaluated with Funnel and Baujat plots. The results of this study provide a quantitative assessment of recovery in chronic stroke in terms of FMA-UE changes in response to non-specific exercise, which can be used as a reference for assessing the relative effect size of future targeted rehabilitation therapies, including those using advanced technology such as robotics, virtual reality or brain-computer interfaces for example.

## 2 Methods

### 2.1 Study design and inclusion criteria

A literature search was performed from the PubMed electronic database for articles published from January 2000 to June 2020. PubMed was selected as the search database for this study due to its comprehensive coverage of peer-reviewed biomedical literature and its indexing of the most relevant journals in neurology and stroke rehabilitation. As a curated and free resource maintained by the National Center for Biotechnology Information (NCBI), PubMed is widely recognized as a gold standard for medical literature searches. The use of PubMed in our study ensured the identification of relevant studies without introducing excessive heterogeneity from less-regulated sources.

The search strategy which is illustrated in the PRISMA flow diagram (Appendix 1) used search terms “Chronic Stroke,” “Rehabilitation care,” and “Fugl-Meyer Upper Extremity Assessment” and aimed at identifying studies that presented upper limb motor status assessments in persons with chronic impairments after stroke who were in the control arm of a clinical trial, and who were treated with a range of general exercises and non-specific physical activity, including motor skills exercises, mobility training, range-of-motion therapy, but excluding activities geared explicitly toward leveraging mechanism of activity-dependent brain plasticity such as mental imagery, mirror therapy, task-specific training, massed practice and constraint-induced movement therapy, and technology-assisted physical activities such as brain stimulation, robotic technology, and virtual reality. The following inclusion criteria identified studies that were included in the current data analysis. First, only studies published from 2000 to June 2020 were included. Second, only investigations reported in an original publication as a full paper were included, excluding abstracts and short communications. Third, study assessments of upper limb motor status needed to include the Fugl-Meyer Assessment of Upper Extremity (FMA-UE). Fourth, the study included a control arm with at least five subjects that received the control rehabilitation therapy provided by the study or general exercise or both. Specifically, for inclusion in this research, a study must have included serial FMA-UE assessments on control patients (a) immediately before and (b) after control-group general exercise therapy. These criteria led to the exclusion of studies that used different assessment methodologies or displayed an absence of a control group. Two clinical experts in Stroke Neurology screened all potential publications of interest independently and reviewed each potential study and resolved disagreements together to ensure that the inclusion criteria were respected.

Overall, the studies identified for inclusion in the data analysis had comparable parameters concerning the reporting of the FMA-UE score, typically reported by an average and standard deviation over the control group. However, the timeline of the assessments obtained from participants and the nature of the intervention on the control group varied across studies. Some studies did not report the standard deviation (SD) for the control group outcomes directly. In such cases, we estimated the SD by either dividing the interquartile range (IQR) by 1.35 (19) or applying the range rule, which converts the sample range into an SD estimate (7).

**Figure 1:**
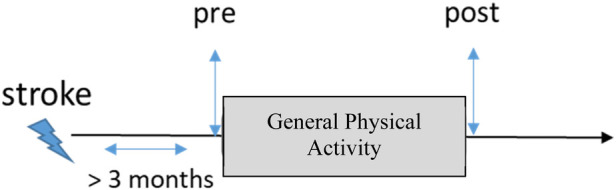
Timeline of evaluation. The control group of the studies included in this study underwent general physical activity. Upper extremity motor status was assessed before the start of the study (i.e., pre) and immediately after (i.e., post).

Figure 1 illustrates the timeline of rehabilitation and assessment in a typical scenario in which the intervention occurs at least 3 months after a stroke. In all included studies, FMA-UE was assessed prior to (i.e., pre) and immediately after (i.e., post) general exercise rehabilitation therapy. Therefore, the serial change ΔFM in terms of FM score was computed as the difference between $FM_{\;pre}$ and $\;FM_{\;post}$. Some studies included additional follow-up assessments that were also recorded in terms of FMA-UE scores. However, to ensure consistency in the current analysis, we did not include those additional FMA-UE scores as part of our data analysis.

### 2.2 Fugl-Meyer assessment of upper extremity (FMA-UE)

Individuals with chronic stroke comprise a heterogeneous population with a wide range of upper extremity motor impairments. To facilitate planning treatment and evaluation of progress in a clinical, research, or community setting, stroke survivors require thorough assessment. While both research and clinical guidelines lack consensus on a primary outcome measure, the FMA-UE (or simply FM) scale of motor impairment is the most used assessment for measuring post-stroke impairment within the research context.

The FMA-UE scale has been used as an inclusion criterion, as the basis for stratifying study subjects based on motor deficit severity, and as an outcome measure for clinical trials. Recent studies (8–12) have compared assessment tools, including the FMA-UE scale, to determine the optimal method to evaluate post-stroke impairment and demonstrated its reliability, validity, and sensitivity to treatment-related change.

The FMA-UE has four subsections: (1) shoulder-arm, (2) wrist, (3) hand, and (4) coordination and speed. They are designed to measure impairment from proximal to distal and from synergistic to fractionated voluntary movements. The four subsections are performed in an ascending numerical order that approximates the sequence of recovery post-stroke. Each of the 33 items that constitute the FM is scored on an ordinal scale: 0 (absent), 1 (partial impairment), or 2 (no impairment), which in sum results in a range of possible scores from 0 to 66.

### 2.3 Longitudinal aggregate data analysis

The data extracted from the results reported in each study allow the effect size and its variance to be computed for each control group. The effect size corresponds to a standardized mean difference (SMD) that quantifies differences between a baseline and a follow-up FMA-UE assessment in standard deviation units. The value for SMD is calculated so that positive values indicate that the group of subjects that received standard rehabilitation care in each study demonstrated improvement over time in terms of FMA-UE score. The SMD is also known as Cohen's *d.* The general formula used to compute SMD and its variance follows:

$$SMD=\;\frac{\bar{FM_{t1}}-\bar{FM_{t2}}}{SD_{\;pooled}}$$

$$SD_{\;pooled}=\;\sqrt{\frac{s_{1}^{2}+s_{2}^{2}}{2}}$$

$$var(SMD)=\frac{n_{t1}+n_{t2}}{n_{t1}\times n_{t2}}+\;\frac{SMD^{2}}{2(n_{t1}+n_{t2})}$$

$$SE(SMD)=\sqrt{\frac{var(SMD)}{\;n_{t1}+n_{t2}}}$$

Where $\bar{FM_{t1}}$ and $\bar{FM_{t2}}$ are the average FMA-UE assessment for the control group at baseline and follow-up, respectively. $SD_{\;pooled}$ is the standard deviation of the serial difference. Here, var(SMD) and SE(SMD) denote the variance and standard error of SMD, respectively. For some studies, it was necessary to derive the standard deviation from the 95% confidence interval or the interquartile range employing appropriate conversion. The data analysis consisted of computing the individual effect size of each of the 20 studies (Table 1).

**Table 1:** Summary statistics of the studies computed in our data analysis, including sample size, average Fugl-Meyer assessment of upper extremity (FM) at baseline and post-standard rehabilitation care, serial change in FM between baseline and post, and effect size in terms of standardized mean difference and standard error.

Study ID	Study name	Sample size	Baseline	Post	Δ FM	Effect size	
			Avg FM	Avg FM		SMD	SE
14	Page et al. (2004) (21)	6	32.55	29.7	−2.85	−0.29	0.91
17	Pang et al. (2006) (22)	30	51.3	52.5	1.2	0.06	0.43
2	Page et al. (2007) (2)	16	35.75	36.75	1	0.1	0.53
7	Stinear et al. (2008) (7)	16	17.6	19.6	2	0.12	0.61
13	Chae (2009) (23)	13	32.3	33.8	1.5	0.13	0.62
20	Housman et al. (2009) (24)	14	18.1	20.3	2.2	0.55	2.10
16	Lin et al. (2009) (25)	20	49.75	51.25	1.5	0.12	0.63
11	Lindenberg et al. (2010) (26)	10	39.8	41	1.2	0.10	0.55
12	Lo et al. (2010) (27)	27	20.3	19.24	−1.06	−0.11	0.64
8	Michielsen et al. (2011) (13)	20	36.4	36.6	0.2	0.01	0.32
15	Line et al. (2010) (33)	17	53.53	54.99	1.46	0.16	0.76
10	Liao et al. (2012) (20)	10	39.6	40.9	1.3	0.11	0.56
19	Reinkensmeyer et al. (2012) (28)	13	22.9	23	0.1	0.01	0.40
3	Klamroth et al. (2014) (3)	35	20.7	23.3	2.6	0.32	1.89
18	Timmermans et al. (2014) (19)	11	53	54	1	0.14	0.64
4	Fleming et al. (2015) (4)	17	37.5	38.1	0.6	0.02	0.36
5	Cacho et al. (2015) (5)	10	34.9	38.3	3.4	0.18	0.73
9	Colomer et al. (2016) (32)	16	9	9.5	0.5	0.16	0.72
1	Levy et al. (2016) (1)	56	37.6	40.7	3.1	0.45	3.39
6	Chen et al. (2019) (6)	11	30.03	27.06	−2.97	−0.14	0.63
Total (all studies, *N* = 20)		368	33.6 ± 12.7	34.5 ± 12.9	0.9 ± 1.66	0.11	0.87

**Table 2:** Summary of studies included in this data analysis regarding study name, sample size, duration between assessments, number of rehabilitation hours, and type of rehabilitation provided.

Study ID	Study name	Sample size	Time elapsed between	Rehabilitation hours	Rehabilitation care provided by the study
			Assessments in weeks		
14	Page et al. (2004) (21)	6	10	0	
17	Pang et al. (2006) (22)	30	19	57	1-h sessions, 3 sessions per week for 19 weeks
2	Page et al. (2007) (2)	16	7	6	2 days per week, in 30-min segments, for 6 weeks
7	Stinear et al. (2008) (7)	16	4	14	30 min per day over 4 weeks
13	Chae (2009) (23)	13	6	18	18 h of therapy over a 6-week period
20	Housman et al. (2009) (24)	14	34	24	24 × 1-h treatment sessions, approximately 3 times per week for 8–9 weeks
16	Lin et al. (2009) (25)	20	3	30	2 h/day, 5 days/week, for 3 weeks
11	Lindenberg et al. (2010) (26)	10	0.5	2.5	
12	Lo et al. (2010) (27)	27	12	0	The usual-care group received customary care available (i.e., medical management, clinic visits as needed, and in some cases rehabilitation services)
8	Michielsen et al. (2011) (13)	20	7	30	Standard occupational therapy treatment that also focused on UE training and included neurodevelopmental techniques, trunk–arm control weight bearing by the affected arm, fine motor tasks practice, and practice on compensatory strategies for daily activities
15	Line et al. (2010) (33)	17	3	30	
10	Liao et al. (2012) (20)	10	4	30	20 training sessions (90–105 min a day, 5 days a week for 4 weeks)
19	Reinkensmeyer et al. (2012) (28)	13	3	24	24 × 1-h treatment sessions, approximately three times per week for 8–9 weeks
3	Klamroth et al. (2014) (3)	35	8	18	8 weeks, three times weekly (total 24 sessions). 1 session (45 min) per day
18	Timmermans et al. (2014) (19)	11	8	32	8 weeks, 4 times/week, twice a day for 30 min
4	Fleming et al. (2015) (4)	17	0.5	24	Each session contained 2 h of SS (sham) immediately prior to 30 min of TST
5	Cacho et al. (2015) (5)	10	2	15	45 min, twice a week, totaling 20 sessions
9	Colomer et al. (2016) (32)	16	8	18	24 sessions, 45 min each (per week) + physical therapy (5 h per week)
1	Levy et al. (2016) (1)	56	6	65	5 days a week for the first 4 weeks and 3 days a week for the next 2 weeks
6	Chen et al. (2019) (6)	11	2	15	Conventional physical and occupational therapy (90 min/session, 5 sessions/week)
Average		18.4 ± 11.4	7.4 ± 7.6	22.6 ± 16.6	

### 2.4 Evaluation of publication bias

We compute a funnel plot (34) to detect publication bias and highlight potential studies where effect sizes are asymmetrically distributed around the weighted average effect size for those studies that have low precision. Precision, in this context, refers to the accuracy of a study's findings. Quantitatively, this is captured by the standard error computed for individual effect sizes. Thus, a study with relatively low precision has a larger standard error than a study with relatively high precision. Egger's test is used to measure the asymmetry quantitively.

### 2.5 Contribution of individual studies

We use a Baujat Plot (35) to detect studies that contribute to the heterogeneity of our analysis. The plot shows the contribution of each study to the overall heterogeneity as measured by Cochran's Q on the horizontal axis and its influence on the pooled effect size on the vertical axis. As we want to assess heterogeneity and the studies contributing to it, all studies on the right side of the plot are important to observe, as this means that they cause much of the heterogeneity. This is even more important when a study contributes much to the overall heterogeneity while, at the same time, not being very influential concerning the overall pooled effect (e.g., because the study had a very small sample size). Therefore, if studies are present on the lower right quarter of the Baujat plot, they ought to be carefully reviewed.

### 2.6 Heterogeneity

The *I*^2^ statistic is utilized to measure heterogeneity between effect sizes. This index expresses the amount of between-study error as a percentage. Alternatively stated, the index measures the heterogeneity in effect sizes not attributable to within-study error/sampling error.

### 2.7 Baseline FM score

We investigate the relationship between the average baseline FMA-UE score and the effect size for each study. The rationale behind this test is that there exists a difference in potential improvement for patients based on the baseline FMA-UE score. In this type of plot, called a bubble chart, the effect sizes from individual studies are plotted on the *y*-axis, and the baseline FMA-UE score is plotted on the *x*-axis. The size of each circle is set proportional to the standard deviation associated with the effect size.

## 3 Results

Table 2 provides a summary of each study included in this data analysis in terms of the number of participants, intensity, and duration of the rehabilitation sessions. The average number of control subjects per study was 18.4 [6–56], and the average time elapsed between FMA-UE assessments was 8.5 ± 9.2 weeks to compute the serial change, i.e., the ΔFM. The average number of rehabilitation hours for the control group was 22.6 [0–65]. Table 1 illustrates the average baseline, follow-up, and ΔFM scores for each study included in our analysis. The pooled total of patients was 368. The average FMA-UE score at baseline and follow-up was 33.6 ± 12.7 [9–53.5] and 34.5 ± 12.9 [9.5–54.9], respectively. This leads to an average ΔFM of 0.9 ± 1.66 points. The non-weighted standardized mean difference (SMD), or effect size, was 0.11 ± 0.87.

Of the 20 studies included in the analysis, 17 reported improvements in ΔFM scores within their control groups, while 3 studies observed a decline, with reported values of −2.85, −1.06, and −2.97 in Page et al. (21), Lo et al. (27), and Chen et al. (6), respectively. The underlying causes of these declines remain unclear, though the absence of structured rehabilitation exercises in two of the studies (21, 27) may have contributed. It is also possible that other confounding factors, such as participant age, baseline impairment, or the type and intensity of physical activity, also played a role. However, because the reported decline reflects group-level averages, it is not possible to assess the influence of these factors without access to individual-level data and information about these potential confounding factors.

### 3.1 Aggregate statistics

**Figure 2:**
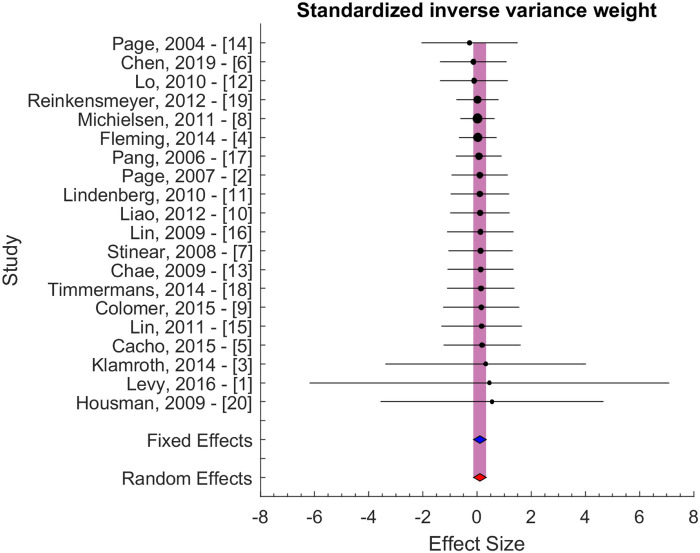
Effect size with standardized inverse variance weight. The blue and red area indicates the fixed and random effect estimates.

The effect size of individual studies was pooled to quantify the overall serial difference between baseline and follow-up assessment for the control group. Figure 2 illustrates the result of the pooling using a standardized inverse variance weight as our principal method. The pooled effect size was computed using a random effects model. Using this model, we assume that heterogeneity, or differences at the study level effect sizes, is the sum of within-study error (e.g., sampling error) and between-study error (e.g., systematic influences on effect sizes). The pooled effect size was 0.11 ± 0.23 (95% CI: −0.05 to 0.26, *p* > 0.05). A significance level of 0.05 was used to evaluate the statistical significance of the computed effect sizes. The pooled effect size did not differ significantly from 0, and thus, we were unable to reject the null hypothesis.

**Figure 3:**
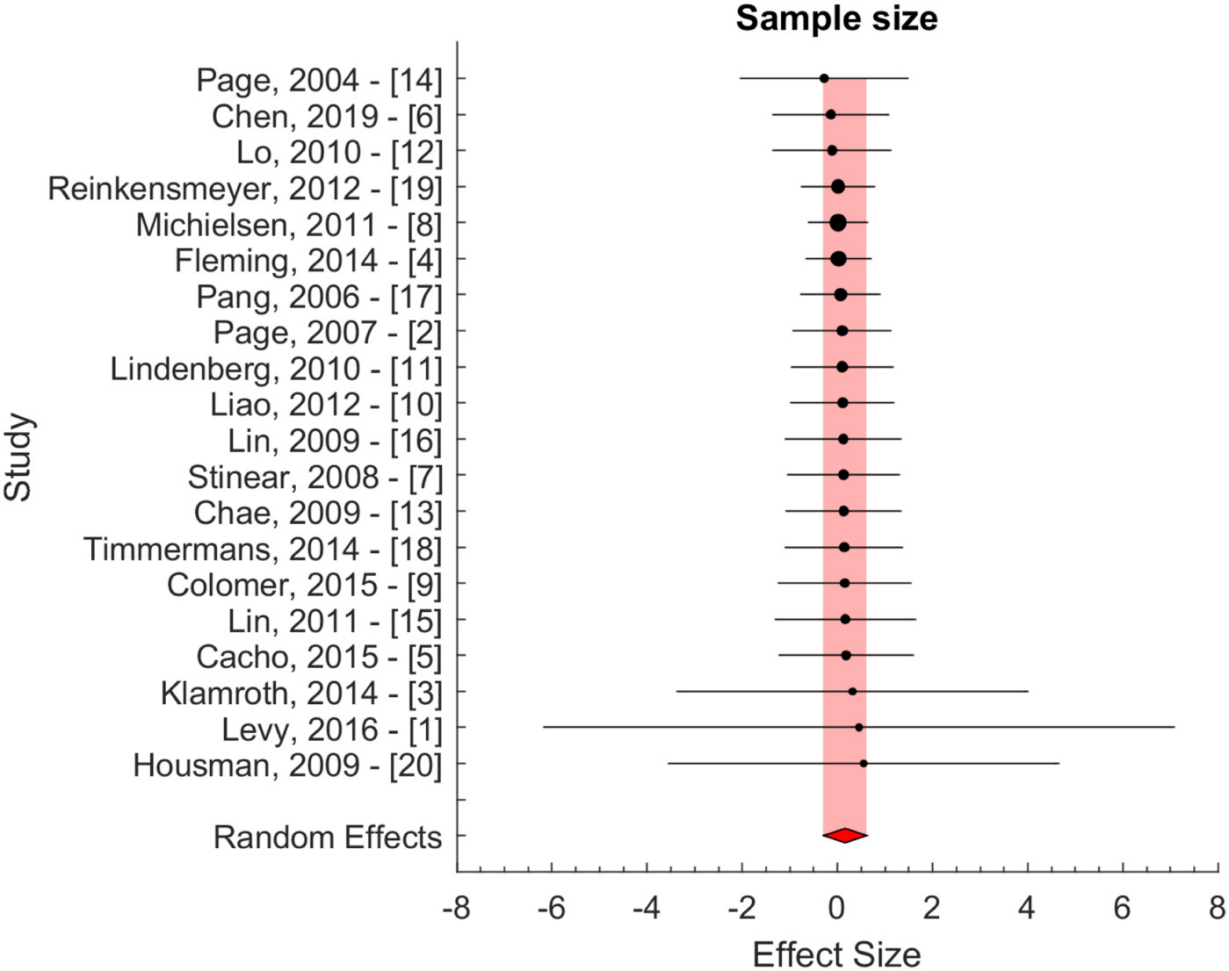
Effect size weighted by sample size. The red area indicates the random effect estimate.

Secondary analyses were performed using inverse variance weight and sample size evaluated potential bias due to differences in variability and sample size (Figure 3). These results confirmed the results of the principal method (Figure 2). The pooled effect sizes were 0.05 ± 0.24 and 0.16 ± 0.45 for the two secondary approaches, i.e., for inverse variance and sample size weighting, respectively.

While none of the control groups included in this study received the main intervention in their respective studies, it is important to note that the control groups of several studies included in our analysis (2, 4, 6, 23, 26) were exposed to some form of sham intervention, which may be associated with a placebo effect. The average ΔFM for these studies was 0.26 ± 1.84, which is lower and not statistically different from the overall average ΔFM of 0.90 ± 1.66 across all studies. Similarly, the pooled effect size for these studies was 0.04 ± 0.11, which is smaller and not statistically different from the overall effect size of 0.11 ± 0.23.

In most meta-analyses, the test usually aims to evaluate the presence and strength of a treatment effect between a control group and an intervention group. The type of data analysis performed here is different in nature as we quantified the effect size based on longitudinal assessments of a given group (instead of changes between groups). Although less common, similar effect sizes have been reported in other medical research areas and are usually referred to as longitudinal data analyses or meta-analyses. To the best of our knowledge, the present study is the first to perform a longitudinal analysis on persons with chronic impairments after stroke by aggregating the control group across studies.

### 3.2 Evaluation of publication bias

**Figure 4:**
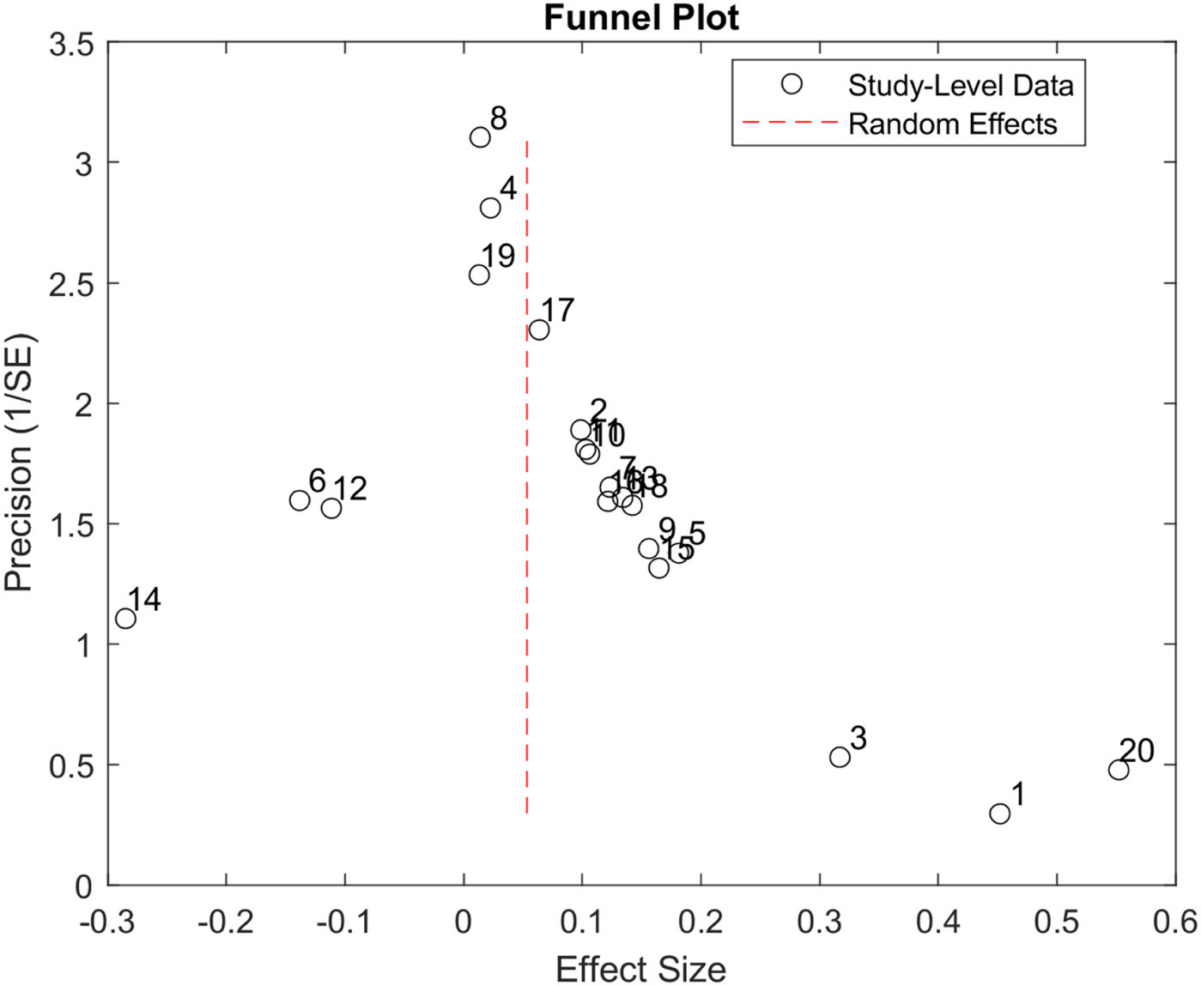
Funnel plot produced using the 20 studies included in our study. The *X*-axis represents the study's effect size, and the *Y*-axis represents the precision of the study. The plot shows that more precise studies tend to aggregate close to the overall effect size of the data analysis.

Figure 4 presents the analysis investigating selection/publication bias using a funnel plot (34). The plot is mostly symmetric. Egger's asymmetry test was not significant, indicating that publication bias, bias due to publication selection in this data analysis, or both were not present.

### 3.3 Contribution of individual studies

**Figure 5:**
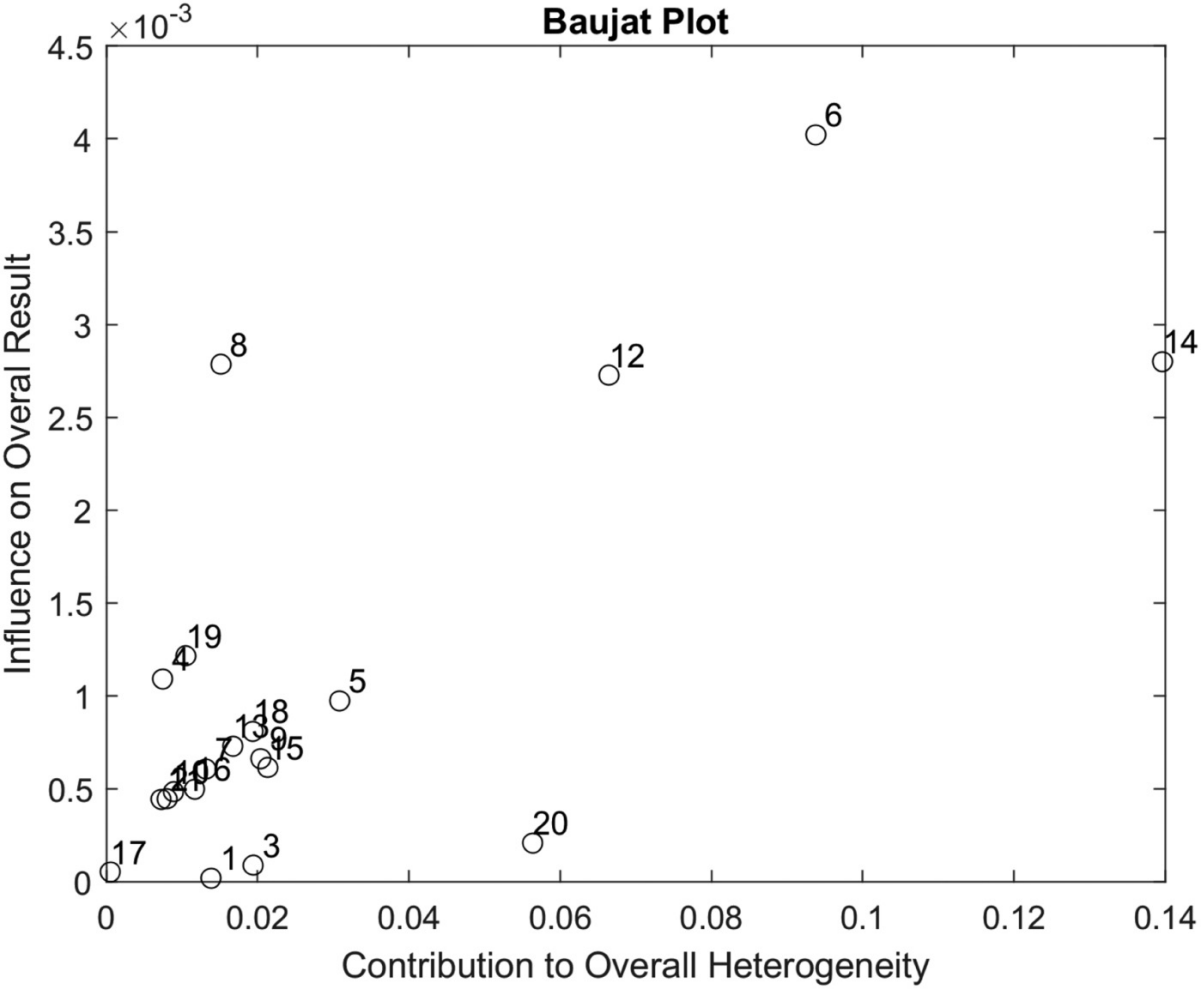
Baujat plot used to assess the contribution of each of the 20 studies included in our data analysis.

Figure 5 presents the Baujat plot for the included studies. None of the studies appear in the lower right quadrant, indicating that no single study substantially contributes to both heterogeneity and influence on the pooled effect size.

### 3.4 Heterogeneity

As a guideline for interpreting the *I*^2^ statistic, Higgins et al. (29) proposed that values of 25%, 50%, and 75% correspond to low, moderate, and high levels of between-study heterogeneity, respectively. In our analysis, *I*^2^ was calculated at 32%, indicating moderate heterogeneity among the included studies.

To explore potential sources of this heterogeneity, we examined whether rehabilitation hours and the time elapsed between assessments were associated with upper extremity motor improvement ΔFM.

A Pearson correlation analysis between rehabilitation hours and effect size yielded a moderate positive correlation (*r* = 0.46, *p* = 0.04), which was statistically significant at the 5% level. This finding suggests that greater rehabilitation exposure is meaningfully associated with larger treatment effects. A similar analysis between rehabilitation hours and ΔFM resulted in a correlation coefficient of *r* = 0.43 with a *p*-value of 0.06. Although this correlation also indicates a moderate positive relationship, the result did not reach conventional statistical significance (*p* > 0.05). These findings are promising but preliminary and require confirmation through further research.

We also assessed whether time elapsed between assessments was associated with outcome. The correlation between time elapsed and effect size was *r* = 0.39, with a *p*-value of 0.09. While this suggests a moderate positive trend, it did not reach statistical significance, indicating that longer follow-up durations may be associated with larger effect sizes, but the evidence remains inconclusive. Finally, the correlation between time elapsed and ΔFM was weak and not statistically significant (*r* = 0.07, *p* = 0.77), suggesting that, in this dataset, the duration between assessments was not meaningfully associated with the magnitude of motor recovery.

### 3.5 Baseline FMA-UE score

**Figure 6:**
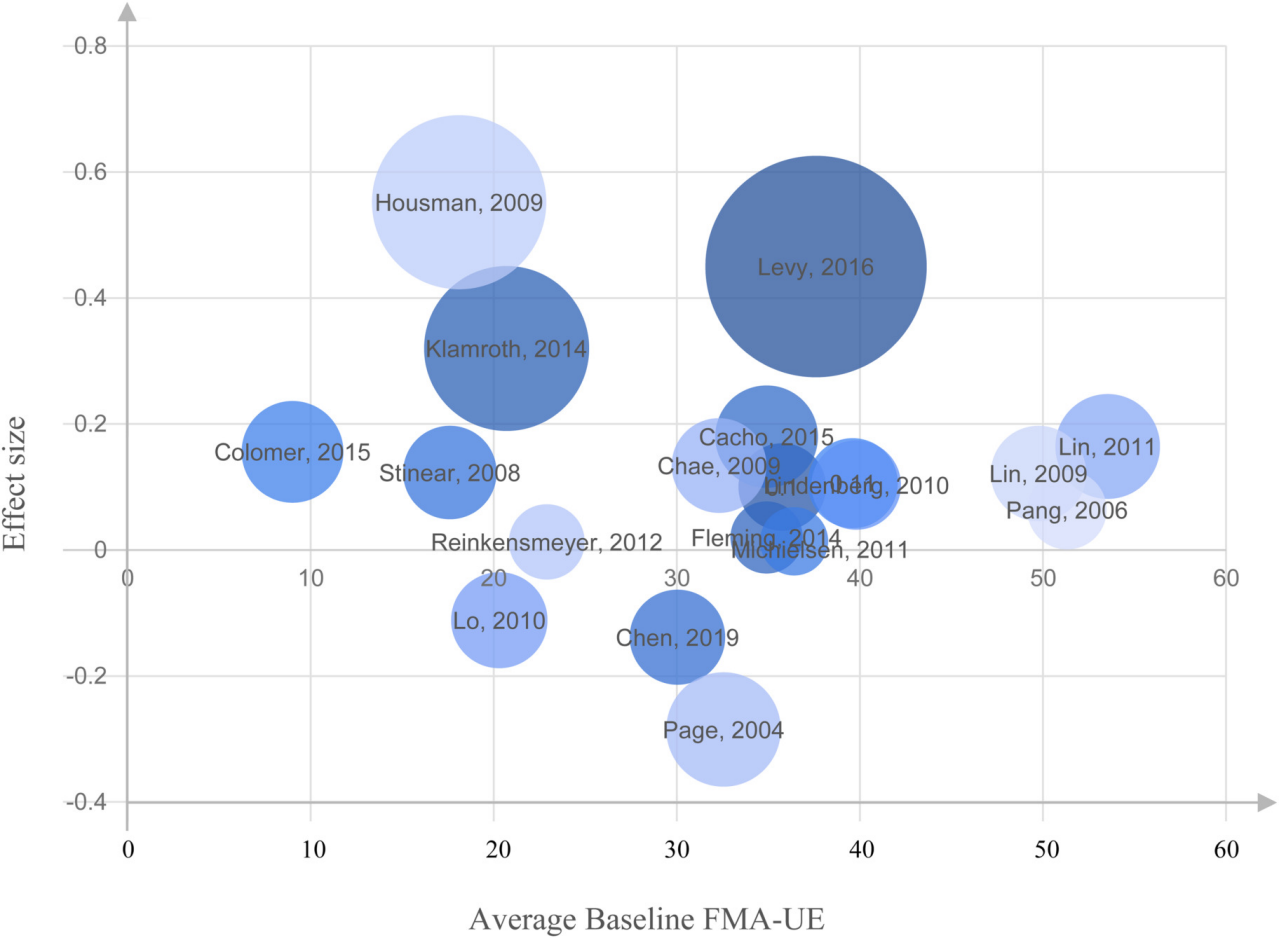
Bubble chart representing the relationship between the average Fugl-Meyer Assessment of Upper Extremity (FMA-UE) at baseline and the effect size for each study. No linear association or correlation was found between the baseline FMA-UE and effect size on the studies included in our analysis.

The relationship between the average baseline FMA-UE score and the effect size is illustrated by a bubble chart in Figure 6, where the average baseline FM is represented on the *X*-axis and the effect size on the *Y*-axis. The size of each circle is set proportional to the standard deviation associated with the effect size. A review of Figure 6 indicates the absence of a linear relationship or correlation between baseline FMA-UE and effect size, demonstrating that the baseline FMA-UE of the patients included in these studies was not a significant bias (nor a predictive factor) of the effect size. Similarly, the correlation coefficient between the baseline FMA-UE and ΔFM yielded weak positive correlation (*r* = 0.11, *p* = 0.65), which was not statistically significant. This suggests that baseline FMA-UE is not a strong predictor of how much improvement occurred in this dataset.

### 3.6 Summary

The effect size computed from serial differences in terms of FMA-UE assessment from a total of 368 patients across 20 trials was 0.11 ± 0.23 (95% CI: −0.05 to 0.26, *p* > 0.05), a value that corresponds to a mean ΔFM of 0.9 ± 1.66 (SD) points over a mean of 7.4 weeks between assessments, and that reflects an average of 22.6 h of rehabilitation therapy. Although small, this estimated average improvement can be hypothesized to be attributable to the training effect, as some persons with chronic impairments after stroke treated with standard rehabilitation care will exhibit an improvement after periods of inactivity. Evaluation with Funnel and Baujat plots indicated a moderate heterogeneity (*I*^2^ of 32%) of the data analysis.

## 4 Discussion

This longitudinal aggregate study supports the concept that persons with chronic impairments after stroke are unlikely to achieve clinically significant motor improvement in upper extremity rehabilitation with general physical activity.

With an average gain of a 0.9-point gain in ΔFM, the observed motor improvements are well below the MCID in the subacute (9–10 points) and chronic (4.25–7.25) phases of stroke recovery. This also indicates that the likelihood of spontaneous recovery occurring after reaching the chronic phase of a stroke is very small (23). The results of our study indicate that the incremental improvements seen within the aggregated control groups could be attributed to the influence of training effects of a subset of activities included as part of general activity. Alternatively, they could result from the Hawthorne Effect, which occurs when a participant's behavior changes because of being observed rather than because of an intervention. The effect size calculated in this analysis is minimal; however, this does not imply that general physical activity is not useful for preventing the accrual of additional disability after stroke. Data suggest that a trajectory of increasing disability with age becomes significantly steeper after stroke (30) and might be mitigated by exercise (31). However, our analysis suggests that general physical activity in chronic stroke rehabilitation yields limited impact on motor recovery outcomes for the upper extremity, potentially affecting community practices and inspiring a reassessment of standard interventions.

The analysis of Cohen's *d* metric provided some general guidelines to interpret the effect size in the context of a meta-analysis and can be considered small, medium, and large when d is above 0.2, 0.5, and 0.8, respectively. The effect size reported in our analysis is 0.11. Therefore, in terms of Cohen's *d*, it is generally considered very small and suggests that the magnitude of the difference between the pre- and post-rehabilitation with the standard of care, or the strength of the relationship between the serial upper extremity rehabilitation scores, is minor.

In general, there is a need for a better/standard definition of standard and usual care. Many persons with chronic impairments after stroke who are chronic in the broad population are not receiving skilled upper extremity rehabilitation services. The participants of the control groups of the above studies received a variable range of exercise and activities and may have been receiving upper extremity rehabilitation. Conventionally, upper extremity rehabilitation is more intervention than what is typically provided to the broad patient population. Overall, this supports the concept that chronic stroke survivors may serve as self-control for upper extremity motor rehabilitation studies if they are not receiving upper extremity rehabilitation at the time of study participation and no other intervention variables are being utilized.

There is also a need for further research addressing the dosing effects of interventions in the chronic stroke population, which may yield improvements in motor function of the upper extremity. However, it is generally believed that patients receiving therapeutic exercise outside of the treatment arm of a controlled trial are massively underdosed and perform many fewer repetitions of functional movements than was previously assumed. Therefore, in the vast majority of cases, it is very unlikely that patients in the chronic phase of stroke will ever achieve the exercise dose in terms of repetitions, duration, frequency, or intensity to experience a change in motor performance outside of the treatment arm. This analysis can also serve as guidance for occupational and physical therapists, supporting utilizing the Fugl-Meyer Assessment of Upper Extremity (FMA-UE) to assess changes in motor impairment when interventions are applied. This also assists in guiding the probable trajectory of motor improvement that may improve in the chronic stroke population.

Limitations of the current analysis include the relatively small number of studies *N* = 20. Also, it was not possible to determine the exact content, dose, and frequency of the exercise programs given to participants in the control arm of the selected studies. Whereas modern ethical standards preclude withholding rehabilitation treatment from persons with chronic impairments after stroke, it also makes it challenging to identify populations engaged in “just exercise” that are not shaped to some extent by a therapeutic framework. The lack of information provided in most papers about the exact nature of the exercise received by the control groups prevented us from running additional heterogeneity assessments or subgroup analyses that could potentially refine and identify differences in effect size. Without standardized reporting, it is also difficult to determine whether observed outcomes are due to the natural course of recovery, the effects of usual care, or variability in physical activity exposure.

In the context of our aggregate analysis, we recognize that each study included may differ in its design quality, execution, and reporting. A risk-of-bias assessment could strengthen the confidence in the clinical recommendations by assessing the trustworthiness of the evidence combined and potentially flag studies that should have been excluded. Our study did not include such assessment which could provide additional insights in future work. The standardized mean difference (SMD) used to obtain the effect size does not account for pre-post correlation. This could be addressed by using standardized mean change (SMC) instead.

Finally, our study considered the late subacute phase and the chronic phase by including patients at the baseline for potential improvements at 3 months post-stroke, which might still be associated with spontaneous recovery. However, our analysis revealed a small effect size despite these confounds.

The key innovation of our study is to perform a longitudinal analysis of persons with chronic impairments after stroke by aggregating the control group across studies (rather than using the intervention group).

## 5 Conclusion

This longitudinal aggregate analysis of upper limb motor status in persons with chronic impairments after stroke computed the effect size when aggregating the control group of each study. The effect size (0.11 ± 0.23) calculated in this data analysis is minimal, indicating that general activity in late subacute and chronic stroke rehabilitation yields limited impact on motor recovery outcomes. However, this does not imply that general physical activity is not useful for preventing the accrual of additional disability after a stroke. Moreover, it does not mean that exercise is ineffective but rather that unsupervised, non-targeted activity is generally not enough on its own to achieve recovery. Our study highlighted that the average number of rehabilitation hours yielded moderate correlation with the effect size and the recovery in motor function, although it was just below the statistical significance threshold of 0.05 for the latter. In addition, the findings are specific to upper extremity function and may not generalize to other domains of stroke rehabilitation.

The small effect size (<0.2) and small motor improvement ΔFM (<1 point) were quantified based on the control arm of the studies included in this analysis. Such limited improvement can be attributed in part to a training effect that is observed when persons with chronic impairments after stroke receive structured rehabilitation therapies after a period of inactivity.

This study can serve as preliminary evidence for a prospective, large-scale meta-analysis that would aim to set a reference effect size for a typical control group associated with chronic strokes. This could be valuable for evaluating future intervention therapies as it could serve as an effect size reference for other interventional studies lacking a contemporaneous control group. Future interventions should exceed the effect size (0.11 ± 0.23) to demonstrate efficacy (32, 33).

## Figures and Tables

**APPENDIX 1:**
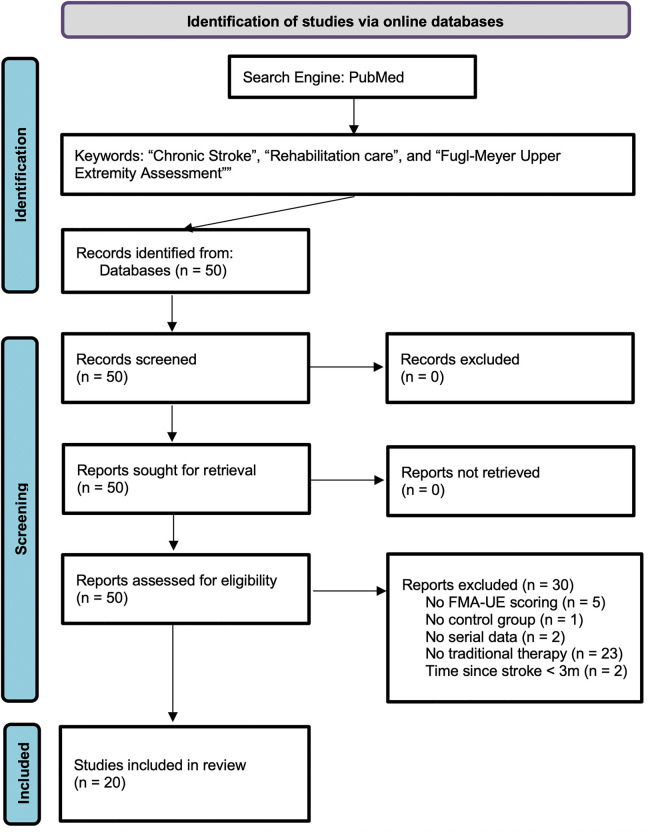
PRISMA flow diagram for the review detailing the search of PubMed database, the number of papers screened, excluded, and ultimately included in this study (36). For more information, visit: http://www.prisma-statement.org/.

## Data Availability

The original contributions presented in the study are included in the article/Supplementary Material, further inquiries can be directed to the corresponding author.
